# Endovascular treatment of epidural arteriovenous fistula associated with sacral arteriovenous malformation: case report

**DOI:** 10.3389/fneur.2024.1326182

**Published:** 2024-02-12

**Authors:** Ahmad Sulaiman Alwahdy

**Affiliations:** Department of Neurology, Interventional Neurology Subdivision, Fatmawati General Central Hospital, Jakarta, Indonesia

**Keywords:** arteriovenous fistula, arteriovenous malformation, embolization, endovascular, epidural, spinal

## Abstract

Spinal epidural arteriovenous fistulas with sacral arteriovenous malformation (AVM) are a rare type of spinal arteriovenous fistulas. There are two varieties of spinal epidural arteriovenous fistulas (SEDAVFs), with type 1 involving intradural venous drainage and type 2 not involving intradural venous drainage. We present a case of transarterial embolization for type 1 SEDAVFs with sacral AVM. Within 8 months, a 14-year-old boy presented with progressively weaker lower extremities and bladder-bowel dysfunction. Magnetic resonance imaging (MRI) of the whole spine revealed thoracic spinal cord congestion, a single dilated flow void running from the lumbosacral area to the conus medullaris, and continuing cranial draining up to the C5 level via the perimedullary vein. Filling of the venous sac through a preferential feeder after embolizing the AVM nidus was performed. After 3 months, the clinical follow-up showed improvement of motoric function, although mild. Endovascular treatment for SEDAVF type 1 might have achieved total obliteration without any procedural complications. Nevertheless, it can be very challenging due to multiple feeders and the presence of an AVM nidus like in this case. However, the most difficult thing in fistula cases is establishing the diagnosis and finding the fistula point. Early treatment is required, due to the fact that longstanding lesions could cause irreversible damage.

## Introduction

Spinal vascular malformations are classified in a variety of ways based on their anatomical location and characteristics. The two most prevalent kinds of spinal vascular malformations are spinal dural arteriovenous fistulas (SDAVFs) and spinal epidural arteriovenous fistulas (SEDAVFs). It is important to distinguish between dural and epidural fistula based on their clinical presentations and preferred approach to therapy, as SEDAVFs are poorly understood and may be mistaken for SDAVFs, according to the literature. The arteriovenous shunt in SEDAVFs is positioned in the epidural space and fills it, whereas the arteriovenous shunt in SDAVFs is located inside the dural sheath of the nerve root and drains directly into an intradural vein without filling the epidural space (1). However, because of the limited number of cases, understanding of the etiology, demography, pathophysiology, and treatment approach for these problems are also limited (2).

Modification of the previous classification of arteriovenous malformations was done by Kim and Spetzler (3) in 2002 and later in 2006. In 2011, Rangel-Castilla categorized extradural AVFs into three types: A, B1, and B2, which may or may not have intradural venous drainage and may or may not have neurological impairments (Table 1) (4). The shunted epidural venous pouch in SEDAVFs can be found ventrally, laterally, or dorsally. To distinguish such venous pouch locations, the interpedicle line was used as a bone structural reference. In ventral and dorsal SEDAVFs, the shunted pouch is located medial to the medial interpedicle line and on the ventral side and dorsal side, respectively, of the spinal canal in the lateral view (1). The shunted pouch in lateral SEDAVFs is placed lateral to the medial interpedicle line on the anterior view.

**Table 1:** Classification of spinal AV shunts reported by Kim and Spetzler (3) and Rangel-Castilla et al. (4).

**Kim and Spetzler (3)**	**Rangel-Castilla et al. (4)**
AVF types:	Extradural AVF
Extradural Intradural Dorsal A: Single arterial feeder B: Multiple arterial feeders Ventral A: Small B: Medium C: Large	A: With intradural venous drainage B1: Without intradural venous drainage B2: Without intradural venous drainage, without neurological deficits
AVM types:	
Extradural-Intradural Intradural Intramedullary Intramedullary-extramedullary Conus medullaris	

AVF, arteriovenous fistula; AVM, arteriovenous malformation.

SEDAVF symptoms at first are frequently vague. Along with symmetrical or asymmetrical sensory symptoms including paresthesia in one or both feet, diffuse or patchy sensory loss can occur. SEDAVFs can present with symptoms secondary to compressive symptoms, or can present secondary to congestive myelopathy and can manifest with both symptoms. Radicular pain can be developed secondary to compressive symptoms. Although micturition and defecation disturbances can happen early in the disease, both often appear in the late stage. Therefore, as a marker for the location of the venous pouch, the pouch's position may also be connected to the symptoms. Vascular engorgement causes a significant mass effect on the nerve root in the ventral or lateral side, which can manifest as benign symptoms such as radiculopathy (5, 6). Nevertheless, in contrast to when the venous pouch position dorsally located, the author believes that compressive symptoms are less frequently presented due to the position being farther from the nerve root.

Lumbosacral fistulas are frequently associated with a fistula in the ventral epidural region that drains into epidural veins as well as a filum terminale vein (7). As a result, when a fistula develops and drains into the intradural venous drainage, it raises the medullary venous pressure by allowing arterial blood to reflux into the intradural veins, causing progressive venous congested myelopathy (VCM) or both symptoms (8, 9). However, the mechanisms of myelopathy can be due to venous hypertension, mechanical compression, and vascular steal effects (2, 10); the elimination of these AVFs is the goal of treatment (11).

The key to treatment in spinal AVFs is the level location and understanding of the angioarchitecture. AV shunts, such as common spinal DAVFs, that are situated in the sacrum or filum terminale can be treated by surgery, an endovascular approach, or both. The chosen course of treatment has been surgery, which has a higher complete obliteration rate (12). Regardless of whether the shunt is dural or epidural, the dilated filum terminale vein, which connects with subarachnoid veins around the cord, can be a sign of where the fistulous point is located in the lumbosacral region (7, 13–15). Nonetheless, due to variations in the angioarchitecture of the shunts, treatment needs to be individualized. A multidisciplinary approach is crucial in the treatment of complex neurovascular diseases, where the combination of endovascular therapy and surgery plays an increasingly significant role.

Although research on sacral EDAVFs is scarce, it is known that the lateral sacral artery (LSA), middle sacral artery, or iliolumbar artery (ILA) are typically responsible for supplying sacral AVFs (16). However, the research mainly consists of case reports, particularly when there is an association with other abnormalities, such as AVM, as in this case, where the fistula point does not go directly to the shunt point but rather feeds the AVM from multiple feeders before draining into epidural veins and the filum terminale vein. Identification and treatment of these lesions are difficult due to the association of abnormalities and the intricate angioarchitecture of these lesions. We were unable to find any case reports with the same abnormalities, to the best of our knowledge. As a result, we describe a case of sacral EDAVFs connected to a sacral AVM that was fed by multiple levels of the LSA and the sacral branch of the ILA.

## Case presentation

A 14-year-old boy experienced left radicular pain that started developing 3 months earlier along with progressive bilateral foot and leg weakness for 8 months. Reviewing his medical history revealed no significant trauma or systemic illness. During a neurological examination, it was noted that the strength of the left and right leg muscles below the knee, dorsi, and plantar flexion had decreased (grade 1) and that there was still slight movement in the iliopsoas, hamstring, and quadricep muscles (grade 2). Pathological reflexes were absent. Spasticity of the lower extremities and muscle wasting at the left and right gastrocnemius were noted. Sensory disturbances below thoracal 12 and decreased pinprick and light touch sensation were detected in the left foot more than the right side. In the last 3 months, his symptoms worsened, he lost control over his bladder and bowel, and became bedridden. At the time of admission, the patient's Aminoff-Logue disability score was G5M3B1, and intermittent urinary catheterization was carried out.

### Radiological findings

**Figure 1:**
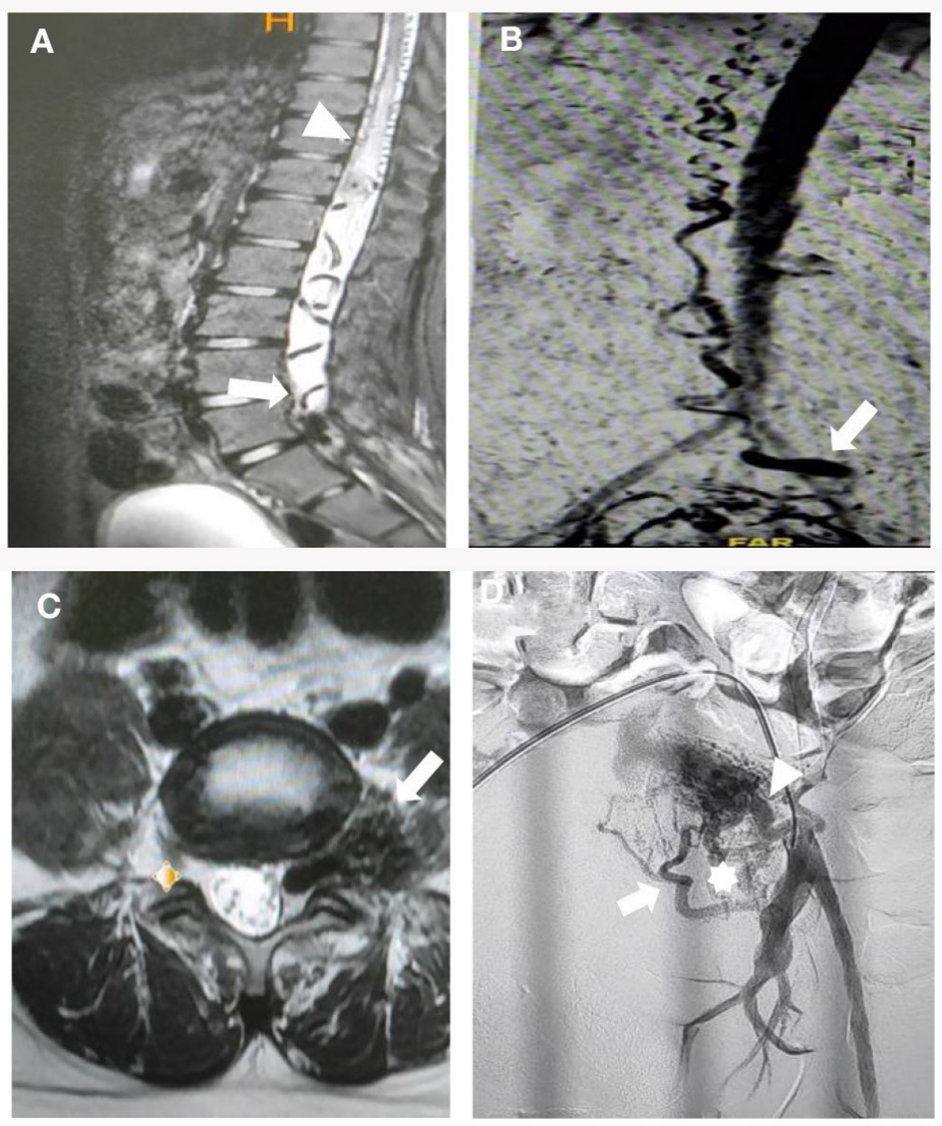
Preoperative multimodal neuroimaging evaluation. **(A)** Diffuse abnormal central-cord T2-weighted image hyperintensity that suggested spinal cord congestion extending from the level of the thoracic region, seen on magnetic resonance imaging (MRI) with contrast, along with a single dilated flow void that ran from the lumbosacral area to the conus medullaris (arrow). **(B)** Contrast-enhanced MR angiography (CE-MRA) showed a venous pouch at the lumbosacral region, with drainage cranially from the filum terminale until the cervical level (arrow). **(C)** Sacral arteriovenous malformation (AVM) was impressed in the left sacral region, with enlargement of the venous pouch before it enters the intradural region (arrow). **(D)** Oblique projection of angiography from the left internal iliac artery demonstrating that a venous pouch was seen at the left L5-S1 region that was associated with sacral AVM that was fed by hypertrophied lateral sacral arteries (LSA) S1 (asterisk), S2 (arrow), and the sacral branch of the iliolumbal artery (ILA) (arrow head).

A contrast-enhanced MRI of the whole spine showed a single dilated flow void extending from the lumbosacral region to the conus medullaris and continuing cranial drainage up to C5 level through the perimedullary vein, as well as diffuse abnormal central-cord T2-weighted image hyperintensity that suggested spinal cord congestion extending from the level of the thoracic region (Figure 1A). Contrast-enhanced MR angiography (CE-MRA) showed perimedullary vein engorgement drainage, cranially from the filum terminale until the cervical level (Figure 1B). The Spinal AVM was impressed in the left sacral region, with enlargement of the venous pouch before it entered the intradural region (Figure 1C). The filum terminale vein must be used for any sacral dural AV shunt draining in the direction of the spinal cord. Therefore, it makes sense to suggest that the presence of an engorged and tortuous intradural filum terminale vein may be a sign of a sacral AV fistula.

A complete diagnostic spinal angiogram, including the iliolumbal artery (ILA), lateral sacral arteries (LSA), and medial sacral artery (MSA) was performed to find the fistula point. From the angiogram, a venous pouch was seen in the left L5-S1 region that was associated with sacral AVM that was fed by multiple arteries with early venous drainage into the filum terminale and drainage to the perimedullary vein cranially, consistent with an SEDAVF type 1 (Figure 1D). After multidisciplinary team meetings and discussion with the patient, transarterial embolization was performed.

### Endovascular treatment

The procedure was carried out under general anesthesia, and the left internal iliac artery was selectively catheterized with a 5-Fr vertebral diagnostic catheter (Terumo, Tokyo, Japan). Each feeder of the AVM from the LSA and ILA was selected using a headway 17 microcatheter (microvention, Aliso Viejo, CA) and a Marathon microcatheter (ev3 Inc.) under fluoroscopic and roadmap guidance. Detachable hypersoft coils (microvention, Aliso Viejo, CA) were placed in additional feeders to reduce collateral inflow from having better penetration and to prevent the liquid materials from refluxing to unwanted locations. The sandwich technique was carried out when onyx 18 (ec3, Irvine, Calif) was injected using a double lumen ballon scepter C microcatheter (microvention, Aliso Viejo, CA). Finally, a guide catheter dextrose 5% push technique was used to inject N-butyl-2-cyanoacrylate-NBCA (Histoacryl; B. Braun, Melsungen, Germany) mixed with Lipiodol (Guerbet, Roissy, France) in a 1:3 ratio through the remaining small, tortuous arterial feeders until the venous pouch and draining vein were filled.

## Discussion

The concomitant presence of multiple spinal vascular diseases in a single patient is very rare. We report a case of sacral EDAVFs supplied by the multiple segments of the LSA and ILA that are idiopathic. Surprisingly, the shunt is not directly toward the drainage vein, which we believe is the filum terminale; however, all the feeders form a nidus that is consistent with the features of AVM in the sacral region. To the best of our knowledge, we could not find any similar cases that have been reported to date in the literature with the same angioarchitecture pattern. Since the feeding artery did not provide a direct shunt to the vein in our case, it cannot be accepted that the fistula-induced myelopathy is caused by a steal phenomenon from the arteries to the fistula. Instead, it is believed that arterialization of the veins increases the venous pressure in the perimedullary venous system, and that the decreased intramedullary arteriovenous pressure gradient causes hypoxic myelopathy.

SEDAVFs have been described as ventral epidural shunts or ventral epidural AVFs in the classification system for spinal arteriovenous shunt diseases (1, 9). The absence of a horizontal T-sign, which is a typical sign of SDAVFs, is not present in SEDAVFs. In addition, SEDAVFs more frequently occur at lumbar spinal levels than SDAVFs and are typically located in the ventral epidural space with multiple feeders. SEDAVFs are brought on by the epidural arterial branches of the ascending cervical, vertebral, intercostal, lumbar, or sacroiliac arteries directly arterializing the epidural venous plexus (1). Sacral EDAVFs are typically located in the ventral epidural space, with the arterialized vein in the root sleeve being distant from the fistula (7).

**Figure 2:**
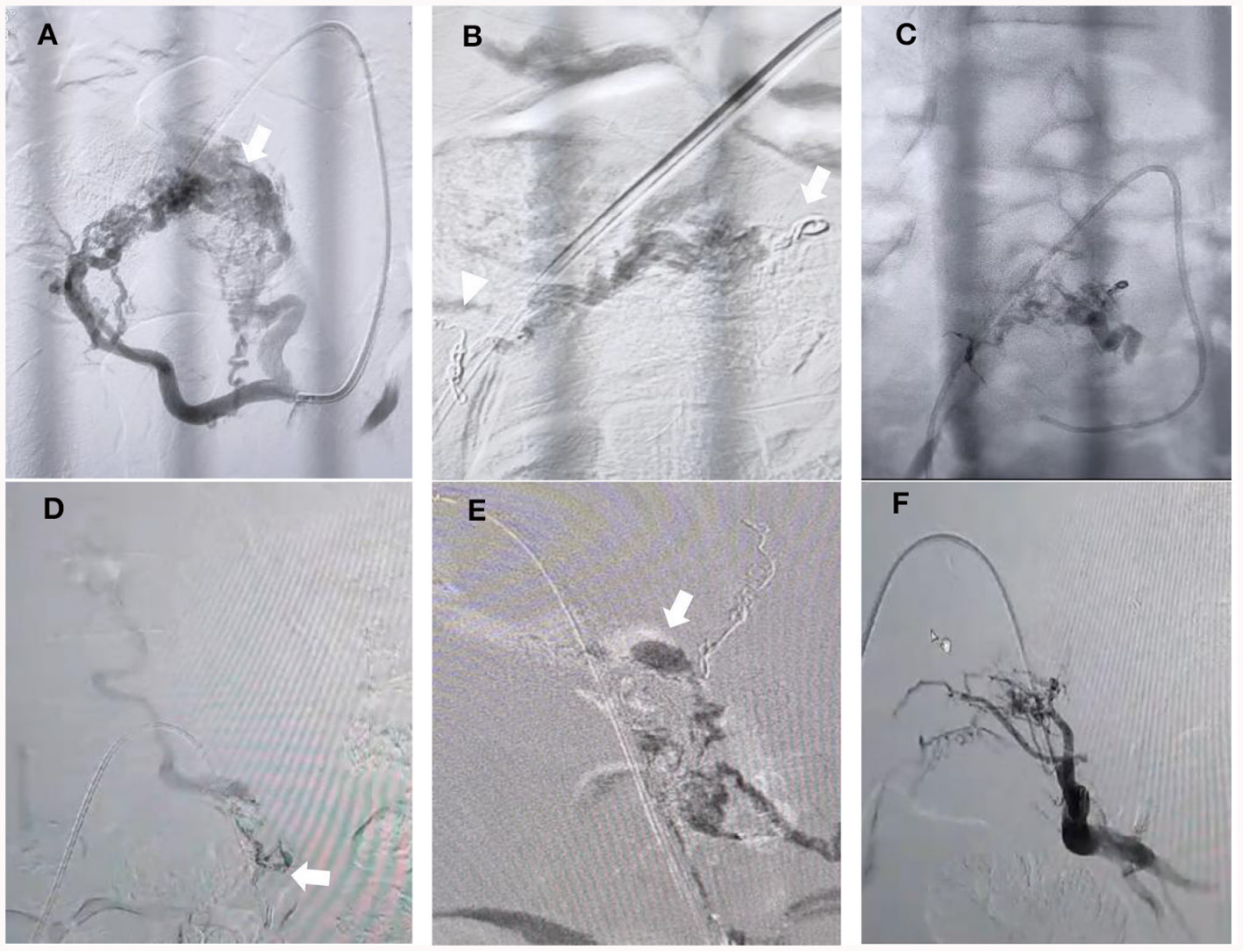
Embolization process. **(A)** Oblique projection of selective angiography from left lateral sacral arteries (LSA) showing that hypertrophied arteries of s2 and s1 fed the nidus AVM, which converged to the shunted pouch in the left ventro-lateral epidural side (arrow). **(B)** Coils in the lumbal branch of the ILA (arrow) and S2 branch of the LSA were performed (arrowhead). **(C)** Final cast after “sandwich technique” Onyx injection was performed over 20 minutes from the main feeder from the S1 branch of the LSA but could not penetrate more distal to the venous pouch. **(D)** Microcatheter injection was performed from the left remaining branch of the LSA but could not advance more distal close to the fistulous side due to the very small and tortuous branch (arrow). **(E)** N-butyl-2-cyanoacrylate (NBCA) injection using the guide catheter dextrose push technique was performed until there was filling of the venous pouch and the draining vein (arrow). **(F)** Final angiography from the internal iliac artery showing complete obliteration of the AVM and fistula.

Contrast-enhanced MR angiography (CE-MRA) provides adequate visualization of the perimedullary and lumbar draining veins and facilitates subsequent digital subtraction angiography (DSA) examinations in the vast majority of cases. The filum terminale is frequently dilated in suspicious lumbosacral region fistulas, which is a helpful sign. However, for accurate fistula localization, DSA remains the gold standard diagnostic tool (14). In our case, the left ventro-lateral epidural side's shunted pouch was fed by a number of feeders from the left sacral branch of the ILA and LSA at the S2 and S1 levels (Figure 2A). The AVFs drained retrogradely through the epidural venous pouch into the filum terminale and drained to the perimedullary vein cranially, consistent with sacral EDAVFs.

Although it has been reported that epidural exposure at the actual fistula site is unnecessary and residual epidural arteriovenous shunting appears to be harmless because it does not produce venous hypertension, a surgical approach to the arterialized venous pouch is risky because the venous pouch is usually located at the ventral epidural space and may require destabilization of vertebral structures to be able to safely manage the malformation (5, 7). So far, the optimal management strategy of SDAVFs has remained unclear (17), especially in our case, due to the fistula associated with the AVM nidus. After multidisciplinary meetings, endovascular treatment was performed, and surgery will be performed if the endovascular approach fails to close the fistula.

The recurrence may have happened when an endovascular approach was chosen, even after the liquid embolic agents partially reached the draining vein. In order to prevent the fistula from recanalizing as a result of the recruitment of more arterial afferents, endovascular treatment of sacral EDAVFs with intradural venous drainage focuses on occluding the epidural venous pouch and the proximal intradural draining vein. For cases with a large epidural venous pouch fed by multiple feeders, complete filling of the embolic materials in the entire venous pouch and draining veins is frequently difficult (6, 18). Nevertheless, complete obliteration can be achieved by a surgical approach; however, concomitant lesions can be challenging (19). During surgery, likely spinal AVM in the common regions requires the inverse approach—the draining vein has to be preserved as much as possible until most or all of the arterial feeders to the nidus are obliterated to prevent rupture (17).

Selecting the optimal liquid embolic agents is crucial, each with its own advantages and disadvantages if the endovascular approach is selected. In earlier reports, particles have the advantages of embolization, particularly prior to operation; however, the agents have a high failure rate over the long term (20). In treating SDAVFs in their study population, Larsen et al. (21) found that liquid embolization using N-butyl-cyanoacrylate (n-BCA) or glue was just as successful as surgical ligation. In fact, when the n-BCA penetrates into the venous side of the nidus, 85% of the fistula is permanently occluded (22). However, when there are multiple artery supplies, it may be challenging to penetrate the venous end with liquid embolic agents, especially if glue is used as it hardens when it comes into contact with blood. Other liquid materials that have been used with success include onyx, squid, and PHIL. Given that each liquid embolic material has its own set of limitations, operator familiarity is very crucial when selecting it. Penetration of the fistula and reflux are still the main challenges with onyx and squid, but forward flow is easier to attain with PHIL. However, PHIL has less radio-opacity than other agents because it is an iodinated contrast-based embolic material (23).

The preferential flow, plug and push, and filling the venous sac techniques have all been mentioned as effective ways to embolize SEDAVFs (24). Nevertheless, when the fistula has a nidus and multiple feeders, it is difficult to know which feeder has dominant flow directed to the pouch due to competing flow from each. Before performing embolization, we placed some coils in the lumbar branch of the ILA and one of the branches of the LSA (S2 level) to decrease the competing flow so as to reduce reflux to an unwanted location and penetrate the feeders that drain dominantly to the venous pouch (Figure 2B). Unfortunately, after embolization from the main trunk of the LSA was performed, the onyx could not penetrate more distal to the venous pouch (Figure 2C). We identified the small branch from the left LSA that fed the fistula, which became more obvious than before and could not be seen at the beginning due to the competing flow. The microcatheter was advanced to the branch but could not advance more distally close to the fistulous side due to the very small and tortuous branch (Figure 2D). Therefore, we decided to inject glue from a microcatheter using the guide catheter dextrose push technique during glue injection to avoid early polymerization and improve distal penetration to the draining vein and venous pouch (Figure 2E). A control angiogram from the internal iliac artery showed that there was no longer an AVM, and fistula drainage was seen (Figure 2F).

Regarding clinical outcomes, after 3 months, improvements in sensoric and motoric strength were noted (grade 2), with an Aminoff-Logue disability score of G4M2B1. The reversibility of the spinal lesions caused by spinal venous congestion myelopathy may be related to complete recovery. When preoperative deficits are mild, clinical outcomes are favorable, particularly in terms of motor and sensory function. Early treatment is therefore necessary because persistent lesions may result in spinal cord damage that is irreversible. However, the limitation of this study is that only one case has been reported, and physicians need to understand the pathophysiology, angiographic, and clinical aspects of the disease in order to determine the optimal treatment for the patient. More similar cases are needed.

## Conclusion

Sacral EDAVFs are uncommon and present a number of diagnostic and treatment challenges. They may have bilateral arterial supplies from the LSA and frequently have multiple feeders, which may affect the results of embolization with liquid embolic agents. Successful management of these lesions necessitates a thorough understanding of the region's variable arterial supply patterns. Misdiagnosis and diagnostic delays are common. In selected patients, prompt diagnosis and treatment are likely to result in better neurological outcomes. To ensure prompt therapy, patients with lower thoracic myelopathy of unknown etiology should have sacral EDAVFs considered in the differential diagnosis. The symptoms can be characterized by the particular anatomy of sacral intradural venous drainage through the filum terminale vein caudally, resulting in venous hypertension in the spinal cord. CE-MRA may be a very useful diagnostic imaging tool to assist in locating the fistula point, especially when a dilated filum terminale is present.

## Data availability statement

The original contributions presented in the study are included in the article/supplementary material, further inquiries can be directed to the corresponding author.

## Ethics statement

Written informed consent was obtained from the individual(s), and minor(s)' legal guardian/next of kin, for the publication of any potentially identifiable images or data included in this article.

## Author contributions

AA: Conceptualization, Data curation, Formal analysis, Funding acquisition, Investigation, Resources, Visualization, Writing – original draft, Writing – review & editing.
